# Spinal Anesthesia Failure Following Wasp Envenomation: A Case Series of Two Patients

**DOI:** 10.1002/ccr3.73637

**Published:** 2026-09-25

**Authors:** Ammir Abuzahra, Mohammad Maraqah, Saja Atawneh, Rasmiya Qawasmi, Mohammad Rabaee, Shaimaa Sweity, Osama Ewidat, Nuha Riyad, Abdulhamid Mujahed

**Affiliations:** ^1^ College of Medicine Hebron University Hebron Palestine; ^2^ Department of Medicine, Faculty of Medicine and Health Sciences An‐Najah National University Nablus Palestine; ^3^ Department of Anesthesia Red Crescent Hospital Hebron Palestine

**Keywords:** case reports, local anesthetics, sodium channels, spinal anesthesia, venoms, wasp stings

## Abstract

Spinal anesthesia is highly effective, and complete failure in healthy patients is rare. Wasp venom contains neuroactive peptides capable of modifying sodium‐channel function and inducing neuroinflammation, but its impact on neuraxial anesthesia has not been previously reported. We describe two healthy ASA I males who developed complete spinal anesthesia failure months after separate wasp stings. Despite correct technique, confirmed CSF flow, and standard doses of hyperbaric bupivacaine, no sensory or motor block occurred in either case, necessitating conversion to general anesthesia. Postoperative evaluations revealed no technical, anatomical, or pharmacologic explanation. The temporal association and biological plausibility raise the possibility that prior wasp envenomation may influence neural sensitivity to local anesthetics through sodium‐channel modulation or inflammatory mechanisms; however, causality cannot be established from these observations. Clinicians should consider recent insect stings when planning neuraxial anesthesia. Further investigation is needed to clarify this potential association.

## Key Clinical Message

Complete spinal anesthesia failure may occur after prior wasp envenomation despite correct technique and standard intrathecal bupivacaine. Although causality cannot be established from two cases, clinicians should consider a history of insect stings when evaluating unexplained neuraxial anesthesia failure and recognize that the proposed venom‐related mechanism remains speculative and unproven.

## 1 Introduction

Spinal anesthesia is a widely used neuraxial technique that provides reliable surgical anesthesia for a variety of procedures, particularly those involving the lower abdomen, pelvis, perineum, and lower extremities. Although generally dependable, spinal anesthesia may occasionally fail because of technical factors, anatomical variation, inadequate drug delivery or spread, or failure of the local anesthetic to act effectively on neural tissue [1]. Reduced effectiveness of local anesthetics has been reported in some patients with connective tissue disorders, including Ehlers–Danlos syndrome Type III, although the underlying mechanism remains uncertain [2].

Interestingly, previous insect envenomation has been proposed as a potential contributor to altered local anesthetic responsiveness. Panditrao et al. reported delayed or incomplete spinal blockade with intrathecal bupivacaine among patients with histories of previous scorpion stings and proposed venom‐related alterations in anesthetic responsiveness as a possible explanation [3]. Wasp venoms also contain biologically active peptides. Pompilidotoxins can modify voltage‐gated sodium‐channel inactivation [4], whereas mastoparan peptides can promote mast‐cell activation and histamine release [5, 6].

We report two otherwise healthy adults who experienced complete spinal anesthesia failure after previous wasp stings. To our knowledge, an association between prior wasp envenomation and subsequent spinal anesthetic failure has not previously been described.

We hypothesize that prior wasp envenomation may have contributed to altered responsiveness to spinal local anesthetics through venom‐related neurophysiologic or inflammatory mechanisms; however, this proposed association remains speculative.

### 1.1 Case 1

A 42‐year‐old healthy male (ASA I) scheduled for inguinal hernia repair had a wasp sting on his forearm 6 months prior. Standard spinal anesthesia was performed at the L3–L4 interspace using a 25‐gauge Quincke spinal needle. Free and continuous cerebrospinal fluid (CSF) flow was obtained before injection. A total of 3 mL of 0.5% hyperbaric bupivacaine was administered intrathecally after confirming correct needle placement. No resistance was encountered during injection, and CSF flow remained evident throughout the procedure. Barbotage was not performed. Despite these findings, the patient showed no sensory or motor block after 10 min. A repeat spinal attempt was performed at a different interspace (L2–L3) using the same technique and anesthetic solution, but yielded identical results. Sensory assessment using pinprick and cold sensation testing demonstrated no detectable sensory blockade at any dermatome level. Motor function was assessed using the modified Bromage scale and remained Grade 0, indicating full lower‐limb movement. He was switched to general anesthesia (GA) uneventfully. Postoperative evaluation and allergy testing were normal. No technical error was identified.

### 1.2 Case 2

A 35‐year‐old healthy male (ASA I) undergoing knee arthroscopy had a wasp sting on his chest 5 months earlier. Spinal anesthesia was performed at the L3–L4 interspace using a 25‐gauge Quincke spinal needle. Free cerebrospinal fluid flow was confirmed before intrathecal injection of 2.5 mL of 0.5% hyperbaric bupivacaine. The injection was smooth and uncomplicated, with continuous CSF return confirming correct needle positioning. Barbotage was not performed. The patient retained full motor and sensory function after 10 min. A second spinal attempt was performed at a different interspace (L2–L3) using the same anesthetic solution and technique, but again produced no sensory or motor blockade. Sensory examination using pinprick and cold sensation testing showed no reduction in sensation, indicating complete absence of sensory blockade. Motor assessment using the modified Bromage scale remained Grade 0, indicating complete preservation of lower‐limb motor function. He was converted to GA and recovered without complications.

The principal clinical and anesthetic characteristics of the two cases are summarized in Table 1.

**TABLE 1:** Comparison of the two cases with spinal block failure after prior wasp envenomation.

Feature	Case 1	Case 2
Age/Sex	42 M	35 M
Surgery	Inguinal hernia repair	Knee arthroscopy
ASA	I	I
Local anesthetic	15 mg hyperbaric bupivacaine	12.5 mg hyperbaric bupivacaine
Response	No block after two attempts	No block after two attempts
Sting history	6 months prior (arm)	5 months prior (chest)
Outcome	Converted to GA	Converted to GA

## 2 Discussion

These two cases share a striking pattern: a complete failure of intrathecal block with standard bupivacaine doses in otherwise healthy patients, temporally associated with a prior wasp sting history. Typical causes of failed spinal anesthesia include technical issues (needle misplacement, inadequate dosing) and patient factors (obesity, spinal pathology), none of which were present [1]. Both blocks were performed by experienced anesthesiologists with correct technique and drug, and both patients were fully cooperative. The incidence of total spinal “failure” in ideal conditions is very low [1]. Therefore, we explored possible physiologic explanations. No intrathecal adjuvants were added in either case because hyperbaric bupivacaine alone is routinely sufficient to achieve surgical anesthesia in healthy adults. The complete absence of both sensory and motor blockade made delayed onset unlikely and suggested complete spinal anesthesia failure rather than inadequate block density. The decision to repeat spinal anesthesia using the same local anesthetic was based on the possibility of an initial technical failure despite apparent correct placement. Repeating the procedure with the standard anesthetic agent represents common clinical practice before abandoning neuraxial anesthesia. However, the repeated absence of any sensory or motor effect strengthened the suspicion that factors beyond technical error were involved.

One possible hypothesis is that previous wasp envenomation contributed to altered neuronal responsiveness to spinal local anesthetics; however, the small number of cases precludes any causal inference. Wasp venoms contain multiple biologically active peptides, including pompilidotoxins that affect voltage‐gated sodium‐channel function [4, 5]. Experimental studies have shown that α‐pompilidotoxin slows sodium‐current inactivation and prolongs neuronal sodium influx [4]. Because local anesthetics such as bupivacaine also act principally through voltage‐gated sodium channels, an interaction is biologically conceivable. Delayed immune‐mediated neurologic complications after wasp stings have been reported [7]. However, no published studies currently demonstrate persistent voltage‐gated sodium‐channel alterations after a remote wasp sting that could alter responsiveness to intrathecal local anesthetics months later; therefore, this proposed mechanism remains speculative.

A second possible mechanism involves venom‐induced inflammatory signaling. Mastoparan peptides in wasp venom can activate mast cells and induce histamine release, as demonstrated in experimental cell models [5, 6]. In theory, inflammatory mediators could alter neural excitability or local anesthetic responsiveness. However, no published evidence currently establishes sustained neuraxial inflammatory changes after a remote wasp sting, and neither patient underwent biomarker or neurophysiological testing to evaluate this hypothesis.

We also considered other explanations, acknowledging limitations. Both patients had no known spinal malformation, infection, or clotting issue that would limit drug spread. Neither patient developed clinical evidence of local anesthetic systemic toxicity. However, the absence of systemic toxicity does not independently exclude problems related to drug preparation, storage, or distribution. Both protocols followed recommendations (bupivacaine dose, positioning, attempts). One could argue subtle technique differences or intervertebral level differences, but the repeated negative result and normal appearance of CSF in all passes make purely technical cause unlikely. The temporal pattern (months after sting) argues against an acute allergic reaction (none occurred perioperatively) but suggests a long‐lasting change. As case reports, causality cannot be proven. In particular, only two cases are presented; this might be coincidental, and many patients are stung by wasps without anesthetic issues. We also lacked laboratory or neurophysiological evidence, such as inflammatory biomarker measurements, serum antibody testing, or nerve‐excitability studies, to support a venom‐related mechanism.

These observations should therefore be interpreted as hypothesis‐generating. The previous scorpion‐envenomation study suggests that a history of envenomation may be associated with altered responses to intrathecal bupivacaine, but it does not establish that the same phenomenon occurs after wasp stings [3]. Experimental evidence that wasp venom peptides can modify sodium‐channel activity and induce histamine release provides limited biological plausibility [4, 5, 6]. Larger observational studies, pharmacological testing, and mechanistic investigations are required before any clinical relationship can be established.

These cases describe an unusual temporal association between prior wasp envenomation and complete failure of spinal anesthesia. Although no technical, anatomical, or pharmacologic explanation was identified, causality cannot be established from two isolated observations. Nevertheless, the known neuroactive properties of wasp venom provide biological plausibility for further investigation. Additional case reports and mechanistic studies are required before any definitive relationship can be confirmed.

## Author Contributions

**Ammir Abuzahra:** conceptualization, investigation, writing – review and editing. **Mohammad Maraqah:** writing – review and editing. **Mohammad Rabaee:** formal analysis. **Saja Atawneh:** resources. **Shaimaa Sweity:** data curation, resources. **Rasmiya Qawasmi:** data curation. **Osama Ewidat:** writing – original draft. **Nuha Riyad:** writing – original draft. **Abdulhamid Mujahed:** project administration, supervision.

## Funding

The authors have nothing to report.

## Consent

Written informed consent was obtained from both patients for publication of this case report and any accompanying clinical details. Copies of the written consents are available for review by the Editor‐in‐Chief of this journal upon reasonable request.

## Conflicts of Interest

The authors declare no conflicts of interest.

## Data Availability

The data supporting the findings of this case series are available from the corresponding author upon reasonable request, including for editorial or peer‐review queries, subject to patient confidentiality. All authors reviewed and approved the final manuscript and agree to be accountable for all aspects of the work.
